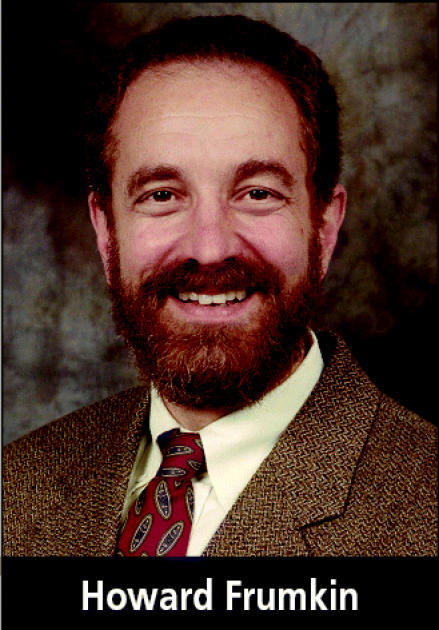# Guest Editorial: Health, Equity, and the Built Environment

**DOI:** 10.1289/ehp.113-a290

**Published:** 2005-05

**Authors:** Howard Frumkin

**Affiliations:** Rollins School of Public Health, Emory University, Atlanta, Georgia, E-mail: medhf@sph.emory.edu

The modern era of environmental health dates from the publication of *Silent Spring* in 1962. In her classic book, Rachel Carson warned of the effects of pesticides on wildlife ecology, invoking a nightmarish die-off of songbirds in the book’s title. However, she also warned of human health effects, both acute and chronic, from liver damage to neurotoxicity to cancer (Carson 1962). In the ensuing decades, environmental health essentially became synonymous with the recognition and control of chemical exposures. Environmental health scientists were toxicologists and epidemiologists, specializing in pesticides, metals, solvents, asbestos, or persistent organic pollutants.

At least two paradigm shifts have revolutionized the field since Rachel Carson’s day. One occurred when environmental health encountered civil rights, forming the environmental justice movement. We are in the midst of the second, as environmental health reunites with architecture and urban planning.

The environmental justice movement coalesced around 1982, when a predominantly African-American community in Warren County, North Carolina, challenged a proposed polychlorinated biphenyl landfill as an act of “environmental racism” (Lee 1992). Early research by sociologist Robert Bullard (1983) found that hazardous waste sites were disproportionately located in African-American communities. Subsequent research documented racial disparities in other hazardous exposures such as industrial plants and bus depots (Bryant and Mohai 1992; Bullard 1990) and even in the enforcement of environmental laws (Lavelle and Coyle 1992).

The environmental justice movement has had a profound effect on environmentalism and on environmental health. It has focused attention on the needs of disenfranchised populations, especially poor people and people of color. In documenting that environmental hazards may target vulnerable populations, it helped draw attention to children, the elderly, people with disabilities, and other groups. It asserted a central role for community perspectives and placed grass-roots leadership at the heart of environmental health advocacy.

A second paradigm shift in environmental health has occurred in recent years: a broadening of focus from the chemical environment to the built environment. Many factors have contributed to this shift. Architectural changes following the oil shocks of the 1970s, especially the construction of “tight buildings,” were found to have health consequences. Rapid urbanization around the world and the sprawling expansion of cities in the United States (Frumkin et al. 2004) gave new meaning—and urgency—to the idea of “urban health.” The obesity epidemic in developed nations called attention to land use and transportation as determinants of physical activity (Saelens et al. 2003). The development of geographic information systems (GIS) facilitated spatial analysis of health problems. Because of these and other factors, environmental health is rediscovering its roots in geography and urban planning (Barton and Tsourou 2000; Corburn 2004).

Each of these trends—the environmental justice movement and the focus on the built environment—has helped transform the environmental health field. Significantly, the two are now converging, as described in this issue of *EHP* (Hood 2005). Disparities in the built environment can be identified in at least five arenas: housing, transportation, food, parks and green spaces, and squalor.

The nation faces a shortage of housing; housing is unaffordable for many poor families; and much of the available housing, especially rental stock, is substandard [Joint Center for Housing Studies (JCHS) 2004]. Substandard housing is clearly bad for health, posing risks that range from lead poisoning to respiratory disease to injuries (Bashir 2002; Krieger and Higgins 2002). Children who live in substandard housing, with such features as rat infestations, leaks, holes in walls and floors, and lack of heat, water, and/or functioning toilets, are at increased risk of emotional disorders (Sharfstein et al. 2001). On the other hand, good housing promotes health and well-being in many ways: providing shelter, serving as “the physical infrastructure for group life,” and providing a secure and rooted sense of home (Fullilove and Fullilove 2000). Poor people and people of color disproportionately reside in substandard housing, a pressing example of health inequities in the built environment.

The term “built environment” conjures images of places—buildings, neighborhoods, parks. But transportation infrastructure forms the connective tissue that links these places together and represents an integral part of the built environment. Equity concerns in transportation take at least two forms. First, certain elements of transportation infrastructure, such as highways and bus depots, are “locally undesirable land uses.” Poor people and people of color disproportionately live near these locations and suffer associated health consequences—the effects of diesel air pollution, noise, injury risks, and ugliness. Second, transportation systems that do not provide poor people with convenient, practical access to employment, medical care, and other necessities undermine their health in numerous ways (Bullard et al. 2004; Schweitzer and Valenzuela 2004). Perhaps most important, the spatial mismatch between where poor people live and where jobs are available, as well as the inability to get to good jobs (Stoll 2005), consigns people to ongoing poverty, a principal predictor of poor health.

There is increasing recognition that the built environment may affect what people eat. In poor neighborhoods where members of minority groups disproportionately live, junk food, soda, and cigarettes are readily available in small markets. Meanwhile, grocery stores that sell fresh foods are scarce and/or expensive (Morland et al. 2002a, 2002b); diabetics have a hard time finding appropriate foods (Horowitz et al. 2004); restaurants are unlikely to serve fresh fruits and vegetables (Edmonds et al. 2001); and liquor stores are common (LaVeist and Wallace 2000). These environmental factors matter; they help explain why people who live in poor neighborhoods eat less healthy diets (Morland et al. 2002a).

Parks and greenspaces represent critically important environmental amenities; contact with nature is highly valued (Kahn 1999), and it offers a range of health benefits (Frumkin 2001). In cities and towns, parks are the principal venue for regular public access to nature. Parks also offer settings for physical activity and social interaction. Racial and ethnic considerations arise in at least two ways. First, racial and ethnic groups vary in their preferences for park features and activities. For example, blacks tend to prefer recreational uses while whites tend to favor land conservation (Payne et al. 2002), and blacks prefer more highly structured and maintained parks, with more facilities, than do whites (Kaplan and Talbot 1988). These differences call for culturally sensitive park design (Rishbeth 2001). Second, members of minority groups in some cities may lack access to parks, trails, and other green spaces (Wolch et al. 2002). Also, a worrisome irony is that urban greenspace increases adjacent residential property values (Crompton 2001). Accordingly, efforts to enhance greenspace access in underprivileged areas of cities could have the unintended effect of raising property values and driving out lower-income residents.

The broken windows theory offers insight into public health. . . . Ultimately, healthy places . . . need to be well designed, well built, attractive, and functional for all people who live, work, learn, and play in them.

The corrosive effects of disorder and squalor in the environment have been widely recognized. Sociologist James Q. Wilson and criminologist George Kelling advanced the “broken windows theory” in 1982, suggesting that the environment sends powerful messages that regulate and release individual behavior: “If a broken window is unrepaired, all the windows will soon be broken. Broken windows are a signal that no one cares” (Wilson and Kelling 1982). Indeed, studies have suggested that sordid environments beget sordid behaviors (Sampson and Groves 1989).

The broken windows theory offers insight into public health. Cohen et al. (2000) found that after controlling for income, race, unemployment, and education, a high “broken windows index” (litter, graffiti, abandoned cars, and blighted housing) independently predicted neighborhood gonorrhea rates. Neighborhood of residence is an important predictor of mortality, an observation that cannot be fully explained by demographic, socioeconomic, lifestyle, and psychosocial factors (Shaw et al. 2000). Part of this effect may well be due to the disorder and squalor of the environment. Poor people and people of color are disproportionately exposed to “broken windows,” another example of a health inequity in the built environment.

In at least five arenas—housing, transportation, food, parks and green spaces, and squalor—environmental justice and the built environment intersect to affect the health of poor people and people of color. Environmental health professionals need to recognize both the scope of the problem and the many opportunities for effective interventions. As Hood (2005) points out, both technical tools (e.g., GIS) and inclusive processes (e.g., community-based participatory research and policy making) can contribute to solutions. Ultimately, healthy places need to be more than free of toxic exposures; they need to be well designed, well built, attractive, and functional for all people who live, work, learn, and play in them.

## Figures and Tables

**Figure:**